# Development of a digital biomarker and intervention for subclinical depression: study protocol for a longitudinal waitlist control study

**DOI:** 10.1186/s40359-023-01215-1

**Published:** 2023-06-22

**Authors:** Gisbert W. Teepe, Yanick X. Lukic, Birgit Kleim, Nicholas C. Jacobson, Fabian Schneider, Prabhakaran Santhanam, Elgar Fleisch, Tobias Kowatsch

**Affiliations:** 1 Centre for Digital Health Interventions, Department of Management, Technology, and Economics, ETH Zurich, Weinbergstrasse 56/56, 8006 Zürich, Switzerland; 2 Department of Psychology, Experimental Psychopathology and Psychotherapy, Binzmühlestrasse 14, Box 8, 8050 Zürich, Switzerland; 3 Department of Psychiatry, Psychotherapy and Psychosomatics, University of Zurich, Lenggstrasse 31, 8032 Zürich, Switzerland; 4 Departments of Biomedical Data Science and Psychiatry, Center for Technology and Behavioral Health, Geisel School of Medicine, Dartmouth College, 46 Centerra Parkway, Lebanon, NH 03766 USA; 5 Centre for Digital Health Intervention, Institute of Technology Management, University of St.Gallen, Dufourstrasse 40a, 9000 St. Gallen, Switzerland

**Keywords:** Depression, Anxiety, Stress, Digital health, Digital biomarker, Breathing training, Conversational agent

## Abstract

**Background:**

Depression remains a global health problem, with its prevalence rising worldwide. Digital biomarkers are increasingly investigated to initiate and tailor scalable interventions targeting depression. Due to the steady influx of new cases, focusing on treatment alone will not suffice; academics and practitioners need to focus on the prevention of depression (i.e., addressing subclinical depression).

**Aim:**

With our study, we aim to (i) develop digital biomarkers for subclinical symptoms of depression, (ii) develop digital biomarkers for severity of subclinical depression, and (iii) investigate the efficacy of a digital intervention in reducing symptoms and severity of subclinical depression.

**Method:**

Participants will interact with the digital intervention BEDDA consisting of a scripted conversational agent, the slow-paced breathing training *Breeze*, and actionable advice for different symptoms. The intervention comprises 30 daily interactions to be completed in less than 45 days. We will collect self-reports regarding mood, agitation, anhedonia (proximal outcomes; first objective), self-reports regarding depression severity (primary distal outcome; second and third objective), anxiety severity (secondary distal outcome; second and third objective), stress (secondary distal outcome; second and third objective), voice, and breathing. A subsample of 25% of the participants will use smartwatches to record physiological data (e.g., heart-rate, heart-rate variability), which will be used in the analyses for all three objectives.

**Discussion:**

Digital voice- and breathing-based biomarkers may improve diagnosis, prevention, and care by enabling an unobtrusive and either complementary or alternative assessment to self-reports. Furthermore, our results may advance our understanding of underlying psychophysiological changes in subclinical depression. Our study also provides further evidence regarding the efficacy of standalone digital health interventions to prevent depression.

*Trial registration* Ethics approval was provided by the Ethics Commission of ETH Zurich (EK-2022-N-31) and the study was registered in the ISRCTN registry (Reference number: ISRCTN38841716, Submission date: 20/08/2022).

**Supplementary Information:**

The online version contains supplementary material available at 10.1186/s40359-023-01215-1.

## Background

Despite effective pharmaceutical, therapeutic, and digital treatments [1], depression remains a global health problem [2] with rising prevalence [3]. Researchers worldwide aim to address this worrisome development by investigating and developing digital interventions, such as smartphone apps, to improve treatment and increase the availability of support. However, given the steady influx of new cases of depression [4], especially due to the Covid-19 pandemic [5, 6], climate change [7], and other world events such as the war in Ukraine, the focus on improving treatment and treatment availability alone may not suffice. It must be complemented by a commitment to improving prevention, i.e., aiding vulnerable individuals who already show weaker symptoms of depression (i.e., subclinical depression) or have biological, social, or environmental predispositions [8, 9].

Subclinical depression, also called minor or subsyndromal depression, is a milder form of depression characterized by less severe and present symptoms. For a diagnosis, an individual needs to report two to four criterion symptoms of depression with at least one of the core symptoms (depressed mood or anhedonia) over a period of two weeks [10, 11]. Evidence shows that many patients with subclinical depression still report persistent depressive symptoms after twelve months when left untreated and that subclinical depression poses a 10–20% risk of deteriorating into a major depressive disorder (MDD) and a 33–50% risk of patients developing moderate functional impairments [10–12].

One approach to improve the prevention and treatment of subclinical and clinical depression is to use digital biomarkers (DBMs) to detect (i.e., screening), diagnose (both within and between clusters of mental diseases), initially tailor treatment (for prevention, especially the tailoring of standalone interventions), and monitor severity and symptoms (for self-monitoring, tracking, or continuous tailoring). Specifically for improving the prevention of depression, DBMs could be used as an early warning system indicating a problem (e.g., when visiting a general practitioner) and continuously monitoring whether changes occur that would require more extensive treatment. While an ideal DBM would cover all of these areas, a validated DBM for one of these functions would warrant significant utility for prevention or treatment. Additionally, for digital health interventions, in particular, DBMs can help to plan treatment before treatment takes place (e.g., when screening) or continuously (e.g., by just-in-time-adaptive interventions [13]). Prior work indicated that initial [14] and just-in-time adaptive tailoring increase the effectiveness of digital health interventions [15].

Regardless of which DBMs are used, they share common advantages and disadvantages. Compared to self-reports, advantages include that DBMs are potentially less burdensome (due to passive collection of data), less difficult to be answered (due to cognitive or disease-specific impairments), not exclusively focused on perceived emotional changes (given that baseline uses physiological or behavioral measurements), less dependent on the location (i.e., remote assessment is possible), less affected by stigma (e.g., when using a clinical assessment), less expensive (especially when a test is carried out by a healthcare professional), and easily embedded within existing interactions (e.g., with a voice assistant) [16–22]. The disadvantages include that DBMs are not as rigorously validated, especially in clinical settings, that large amounts of data are needed to develop the models, and that DBMs can be affected by biases due to race, gender, and age [22]. Henrich et al. [23] described the selectiveness of samples used and reported that most studies rely on white, educated, industrialized, rich, democratic (WEIRD) cohorts to infer cognitive or behavioral changes. Hruschka et al. [24] report that despite these findings, most work still relies on WEIRD populations.

A propitious DBM for subclinical and clinical depression could be speech. One reason is that speech, compared to self-reported behavior or feelings, may be less affected by population biases [22]. Researchers and scientists have long observed that speech is affected by depression [16, 22]. Among the first was Emil Kraepelin, the founder of modern psychiatry, who described his findings regarding voice in depressed individuals in 1921 as follows: “patients speak in a low voice, slowly, hesitatingly, monotonously, sometimes stuttering, whispering, try several times before they bring out a word, become mute in the middle of a sentence” [25]. More recent work laid the theoretical foundation for the utility of speech as a DBM by theorizing speech as a sensitive output system [26] involving cognitive planning, muscular actions, and the respiratory tract and the finding that emotional affect influences speech [27]. Two systematic reviews [16, 22] summarized the evidence researchers have found regarding voice as a diagnostic or prognostic DBM for depression. They also summarize the work investigating which features of the voice (e.g., prosodic features) are most significantly affected by depression and, in the case of Low et al. [22], other mental health diseases. The authors of these two reviews also highlighted speech’s unique advantages as a DBM. These advantages include that speech encompasses a broad range of behaviors, that widely available signal recording technologies simplify data collection (such as smartphones, voice assistants, and voice-activated navigation systems), and that frameworks for feature extraction and advanced machine learning have become easier to use [16, 22, 28]. However, the authors also discussed limitations and necessary future work for developing speech as a DBM. The reviews found that for the development of voice-based DBMs, mostly lengthy therapy sessions served as training data [16, 22]. Low et al. [22] also reported that 32% of the studies used the AVEC (Audio/Visual Emotion Challenge Workshop) databases to train and test different models and that few studies used held-out test sets, with the ones using this type of validation reporting scores ranging from close to chance to high scores (highest reported F1-score = 0.95, however, this score was only achieved in an all-female population). Cummins et al. [16] separated the reviewed studies along different aims: classification of depression presence, classification of depression severity, or regression of depression score level. The maximal accuracy for classification of the presence of depression reported was 96% (for female speakers only), with maximal sensitivity reported as 0.98 (for female speakers only) and maximal specificity reported as 0.94 (for female speakers only). The maximal accuracy of classification for depression severity reported was 79%, with maximal sensitivity reported as 0.88 and maximal specificity reported as 0.77. For regression of depression score level, only studies using different versions of the AVEC database were reviewed. While the comparison between the datasets of different years may produce errors, as mentioned by Cumins et al. [16] the authors report root-mean-square deviations between 7.71 (2014 AVEC) and 12.01 (2013 AVEC). Additionally, it remains unclear to what degree these voice-based DBMs were validated in clinical practice (following the V3 framework from Goldsack et al. [29]) even if the data was collected in a clinical setting. Due to these remaining open questions and potential improvements but also the high potential, we aim to investigate voice as a symptom and severity DBM for subclinical depression.

A less described but potentially highly relevant DBM for depression could be breathing sounds. To the best of our knowledge, breathing sounds have not been investigated as DBMs. However, related work reported that depression affects the brain and physiological structures that have been associated with breathing [30–32]. Depression is associated with poorer lung function [33]. Furthermore, depression and prolonged stress can alter the fraction of exhaled nitric oxide and lung functioning [34]. Additionally, slow-paced breathing interventions that aim to reduce breaths per minute have been shown to improve mood in patients with major depressive disorder [35] and undergraduate psychology students [36]. The advantage of a breathing-based DBM would be that breathing sounds contain less sensitive information than speech and that breathing could be recorded frequently as it occurs several times per minute.

We aim to use breathing sounds as a DBM since slow-paced breathing interventions aiming to slow breathing voluntarily are profoundly intermingled with cognitive aspects of meditation and therapeutic techniques such as biofeedback, progressive relaxation, or autogenic training [37]. Additionally to the development of a DBM, we are also interested in how efficient a slow-paced breathing training in the field is. Related work has shown positive effects of slow-paced breathing interventions on general health status (such as wellness, relaxation, and stress reduction), depression [35], and symptoms of depression such as mood [36]. The positive effects of slowed breathing have led to the development of various breathing guidance apps [38]. However, similar to other digital health interventions, digital slow-paced breathing interventions show substantial declines in adherence after several days [38]. One discussed approach to increasing adherence while maintaining the positive effects of slow-paced breathing is to use biofeedback and gamification. To this end, we described and evaluated the digital slow-paced breathing training *Breeze* in a lab study [39]. However, further evidence is needed to what extent such a digital gamified slow-paced breathing training can reduce symptoms and severity of subclinical depression in the field.

In addition to these voice- and breathing-based DBMs, physiological (e.g., heart-rate, heart-rate variability, skin temperature) and behavioral (physical activity, sleep patterns, location entropy as a surrogate for activity) DBMs for depression have been summarized in a recent systematic review [40]. These physiological and behavioral measurements may serve as complementary or alternative measurements for depression and may be used in addition to self-reported symptoms and cognitive changes. They also provide information regarding physiological changes, such as heart-rate, that may impact voice and breathing. Smartwatches are a commonly used tool to collect both physiological and behavioral data. Additionally, while evidence for the potential of physical activity as both a DBM [41] and a treatment component (e.g., as behavioral activation in CBT) [42] exists, further evidence is needed to investigate whether different symptoms (e.g., mood compared to agitation) are affected equally by increased physical activity.

Due to these advantages of DBMs and the efforts of the scientific community to develop DBMs, we expected to find DBMs already in use by popular apps addressing depression. However, our review of real-world apps for the prevention and treatment of depression available in the Apple App and Google Play Store revealed almost all reviewed apps did not use any DBMs to measure outcomes but relied almost exclusively on self-reports [43]. This observed limited usage of DBMs may partly be due to these markers’ lack of real-world practicability. Privacy concerns could be a reason for the limited use of DBMs due to the need to passively collect data over extended periods without individual interaction [21]. Therefore, alternatives such as only collecting data while users actively interact with a digital health intervention must be investigated. Examples of such data collections are breathing sounds captured while users perform slow-paced breathing training, recordings of voice commands given to a voice assistant, or reaction times of interactions with a conversational agent. Finally, most studies reported in the systematic reviews have used severity assessments such as the PHQ-9 [44] or Beck depression inventory (BDI-II) [45] to develop DBMs. Most other studies have focused on developing DBMs for mood, and less research exists for other relevant symptoms such as agitation or anhedonia.

As summarized by the different reviews [16, 22], substantial work has investigated DBMs for clinical severity of depression. However, it seems especially difficult to categorize patients into low or high-level depression with existing clinical instruments [46]. Additionally, patients with modest levels of depression consulted in clinical settings show the highest chance of misidentification due to the time-consuming process of diagnosing the illness and that not all depressed patients outwardly express emotional symptoms such as sadness or hopelessness [46, 47]. Extreme levels of depression may be easily identifiable using voice as clinicians already use this as an indicator [16]. Therefore, we aim to develop DBMs that detect subclinical depressive symptoms and subclinical depression severity. Following the logic, if a patient is severely impaired by his depression, DBMs may not provide any additional information already evident due to the substantial impairments.

To address these challenges and contribute to the efforts to develop speech- and breathing-based DBMs, we designed and implemented the digital intervention called “On a journey to feel a little better. Or BEDDA” (BEDDA). To improve daily reported symptoms and biweekly reported severity, we implemented intervention components observed to be effective in related work, namely the slow-paced breathing training Breeze [39, 48–50] and a conversational agent [51–54]. Additionally, participants will receive daily short, actionable advice [55–57]. We aim to use BEDDA in a 30-day waitlist control field study to collect different data to address three main objectives.

### Study objectives

The first objective of our study is to develop voice- and breathing base DBMs for mood, agitation, and anhedonia in a range of subclinical depression severity. With our second objective, we aim to develop DBMs for the severity within a subclinical range for depression. Our third and last objective is to investigate the efficacy of BEDDA consisting of the three intervention components, Breeze, a conversational agent, and daily short, actionable advice.

## Methods

We plan a longitudinal waitlist-control field study to address the objectives outlined above. For this purpose, we are developing the smartphone app “On a journey to feel a little better. Or BEDDA.” (BEDDA), which can collect the required data. The three main intervention components of the BEDDA app follow the talk-and-tools paradigm [58]. BEDDA contains two tools (Breeze and daily actionable advice, i.e., daily wisdom) and one talk element (conversation agent). We designed these three elements in line with our three research objectives: (1) Breeze to collect breathing sounds (objectives 1 and 2) and improve symptoms and severity of subclinical depression, (2) daily actionable advice to improve symptoms and severity of subclinical depression, and (3) a socially-oriented conversational agent guiding through the intervention by presenting the story, setting the goal, and presenting gamification elements to increase adherence and efficacy (objectives 1, 2, and 3).

### Conceptual model

**Fig. 1:**
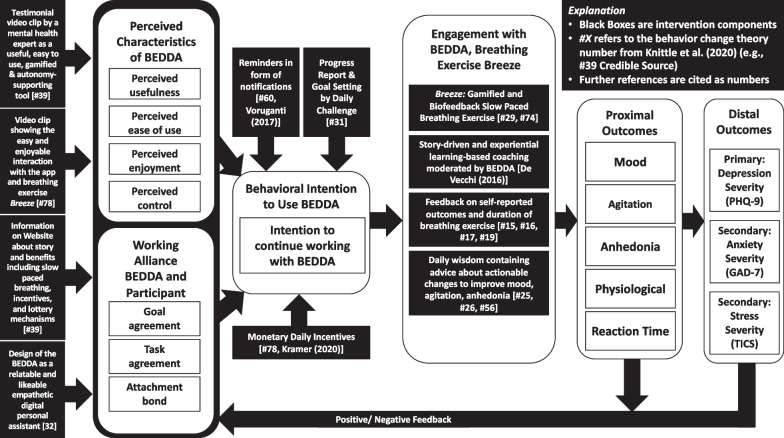
Conceptual model. Conceptual design of the intervention. Intervention components with a number (#) are taken from Knittle [70]. For example, #39 corresponds to the intervention component number 39,“Credible Source” in Knittle, Heino [70]. The remaining intervention components are derived from Vorganti et al. [98], Kramer et al. [73], and De Vecchi et al. [99]

Figure 1 outlines the developed conceptual model of the BEDDA study. This resulting model aims to trigger a causal chain using different intervention components. The intervention components aiming at increasing the engagement of the participants with BEDDA are the black boxes pointing toward “Perceived Characteristics of BEDDA”, “Working Alliance between Participant and CA”, and “Behavioral Intention to Use BEDDA” in Fig. 1. The intervention components that aim to improve the proximal outcomes (mood, agitation, anhedonia) and, in turn, the primary distal outcomes (subclinical depression) as well as secondary distal outcomes (subclinical anxiety) are described in the black boxes pointing toward “Proximal Outcomes” in Fig. 1. For an explanation regarding proximal and distal outcomes of digital interventions, see [13, 59]. In the following sections, we will briefly outline the intervention components we designed to influence the behavioral intention to use BEDDA and improve the proximal and distal outcomes in more detail.

#### Behavioral intention to use the BEDDA app

To increase the “Behavioral intention to use and continue to use BEDDA”, we use the components “Perceived Characteristics of BEDDA” and “Working Alliance Between the Participant and CA”. Related work covering information systems and technology acceptance research [60–63], working alliance [64, 65] linked to conversational agents [52, 66–68], and behavior change theory [69, 70] theoretically informed these components. In addition to increasing the “Behavioral intention to use and continue to use BEDDA”, we will use reminders, progress reports, goal setting by daily challenge, and monetary daily incentives.

#### Smartphone-based biofeedback breathing training Breeze

**Fig. 2:**
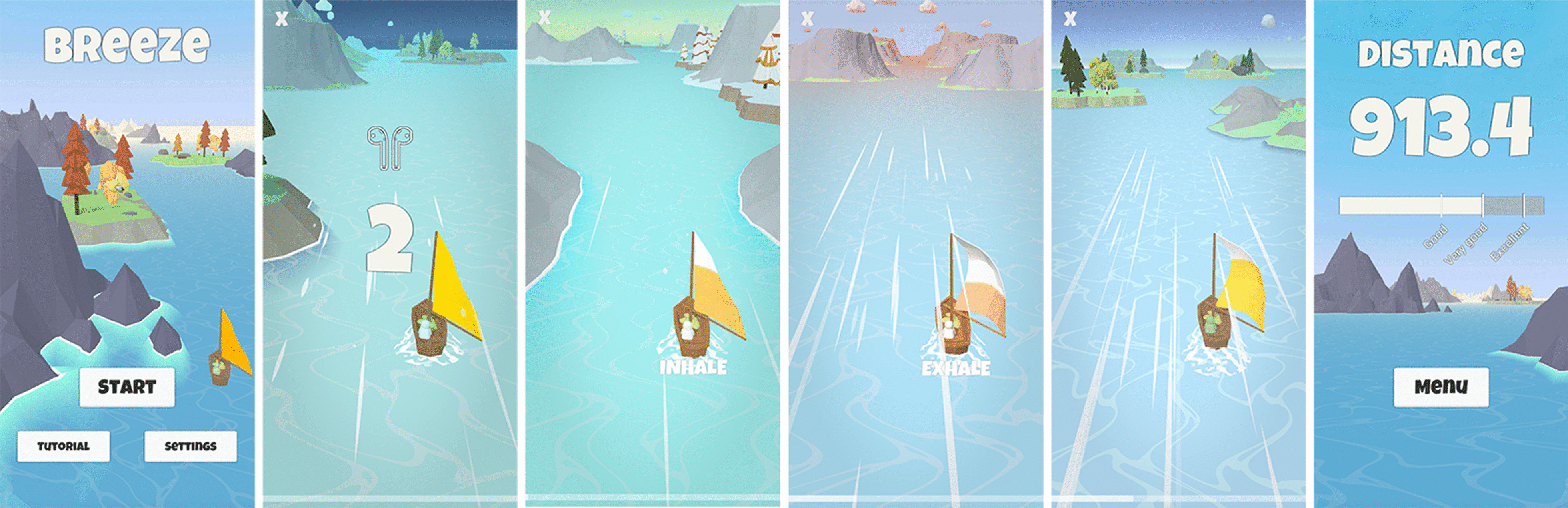
Breeze session. An entire session of Breeze (starting from left): start screen, starting voice commands, countdown, inhalation phase, exhalation phase, ending voice commands, and final screen

Our group initially developed the smartphone-based biofeedback breathing training Breeze and further adapted it to address the objectives of this study [48–50, 71]. Breeze uses the smartphone's microphone to continuously detect breathing phases in real-time (i.e., inhalations, exhalations, and pauses between inhalations and exhalations). This detection is, in turn, used to trigger a gamified biofeedback-guided breathing training visualization. This gamified biofeedback is illustrated as a sailing boat moving down a river, which speeds up when the user performs slow-paced breathing correctly (Fig. 2). This targets experiential outcomes [62] in addition to instrumental outcomes of psychological wellbeing and heart-rate variably (HRV) [72]. The standard configuration of Breeze guides users to breathe with six breaths per minute, which can, however, be adjusted for untrained or well-trained individuals in a specific range safeguarding for too fast or too slow breathing. The gamified breathing training visualization of Breeze has been shown to increase the experiential value [50] compared to a standard breathing training visualization. Furthermore, it has been demonstrated that Breeze effectively increases HRV [49]. Breeze will be explained in the story presented by BEDDA as one of the intervention components.

#### Conversational agent BEDDA

**Fig. 3:**
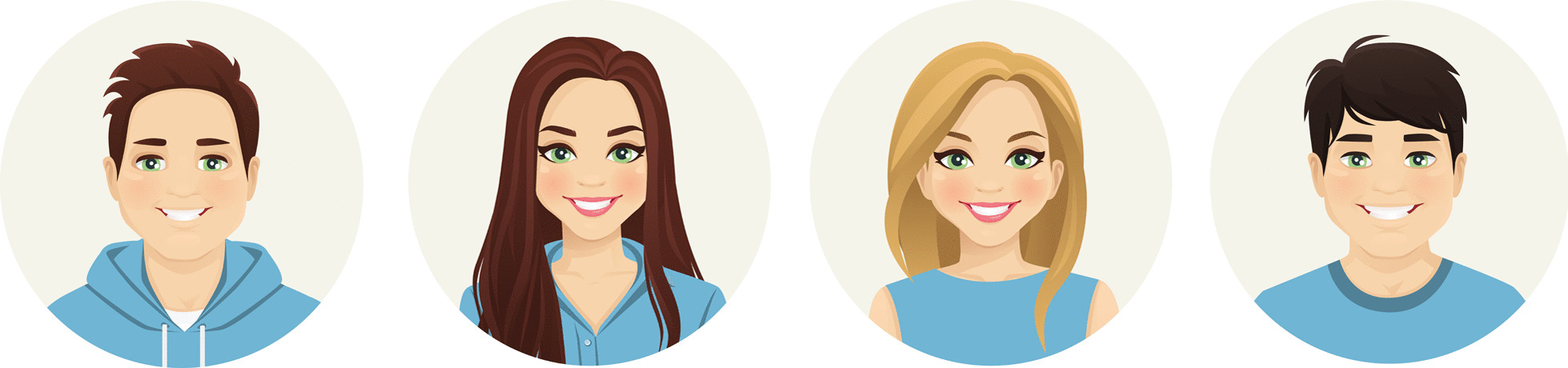
BEDDA Avatars. Conversational agent avatars used in the study. Participants can choose one of the four avatars to represent BEDDA at the beginning of the study. Modified images licensed via ETH Zurich Adobe Creative Cloud License

Analogue to the previous [53, 73–77] and ongoing work of the project team, the intervention will involve a text-based conversational agent (CA) called BEDDA. The participant will choose one of four avatars representing BEDDA (Fig. 3). Like other conversational agents, BEDDA aims to imitate a conversation with a human being [78]. BEDDA will provide a general introduction to the study, explain, and move the story along, collect responses to self-reported questions, and motivate the participant to continue interacting with the intervention. BEDDA will rely on scripted answers to increase simplicity and minimize the risk of harm. No free-text entries will be used except for variables such as the nickname, demographic information, or feedback regarding what to improve in a future intervention version. The study team developed the logic of the BEDDA, and the responses (i.e., conversational turns) are scripted, fully transparent, and traceable. BEDDA will also provide feedback on the self-reported outcomes (self-efficacy, [79]) at the end of the journey.

#### Daily wisdom

The daily wisdom (i.e., short, actionable advice) are actionable tips related to the symptom measured that day. The participant can receive this daily wisdom at the end of each daily interaction on any given day or ignore it for the moment but review it later at any point in the app. We implemented this option of still being able to assess the daily wisdom at a later point to prevent inducing stress on the participants. The daily wisdom is presented as a non-binding and noncommittal option they may want to try at any time. It is designed to be easily implemented into the daily routine and serves to (1) increase the efficacy of the intervention [55]; (2) target improvements in self-confidence and self-efficacy [56]; (3) contribute to the intervention's intensity, which is linked to improving efficacy [57]; and (4) provide an additional measurement for engagement. The list of different daily wisdom can be found in Additional file 1.

#### Gamification and storytelling

The conversational agent named BEDDA will present the story in which BEDDA and the participant go on a treasure hunt. Since BEDDA's sailboat cannot be powered and steered by BEDDA alone, BEDDA asks the participant to power the boat by providing wind energy. BEDDA goes on to explain that at the end of their journey, when they collected 30 keys, a magic chest opens and the coins in it can be collected. BEDDA also explains that the magic chest has another magical power. Provided that the participant manages to collect all keys in less than 40 days, they have the chance to win even further coins (gamification—challenge [70]) or keep a smartwatch received at the beginning of the study (smartwatch group only). Second, if the participant needs more than 45 days to find all keys, the chest loses its magical power, and all gold coins disappear (gamification—challenge [70]). We included this time constraint due to the timescale of this study and to further motivate the participants.

**Fig. 4:**
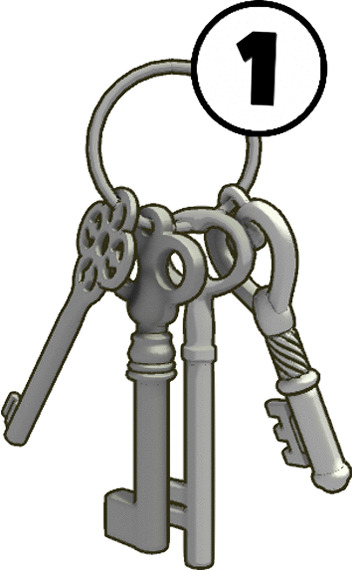
Keys. Graphic illustration of the number of keys collected in the study. The number is increased after each interaction with Breeze. Helen Galliker created images specifically for this study as part of her employment at the Center for Digital Health Interventions. The Center for Digital Health Interventions holds the copywriter to all images

**Fig. 5:**
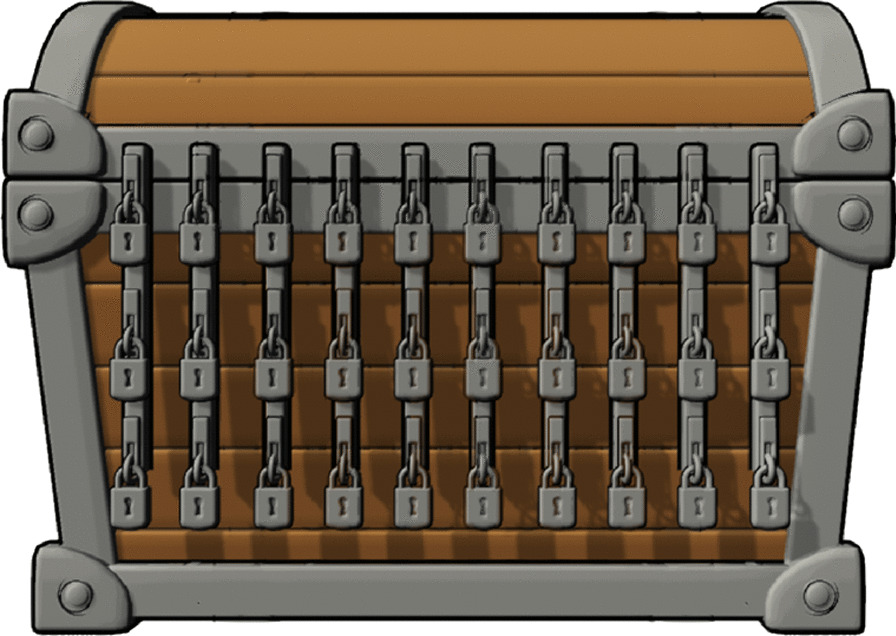
Treasure. The chest with 30 locks the participant aims to open. Helen Galliker created images specifically for this study as part of her employment at the Center for Digital Health Interventions. The Center for Digital Health Interventions holds the copywriter to all images

**Fig. 6:**
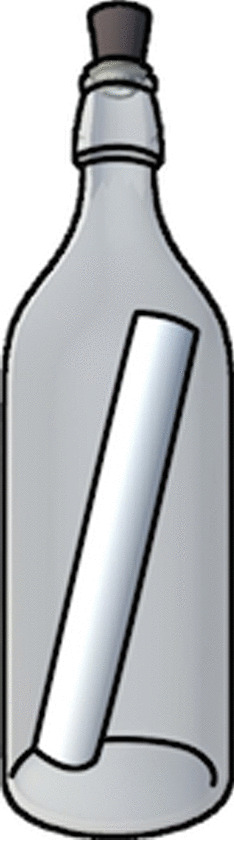
Bottle. Illustration of the bottle in which the daily advice and the keys are found. Helen Galliker created images specifically for this study as part of her employment at the Center for Digital Health Interventions. The Center for Digital Health Interventions holds the copywriter to all images

**Fig. 7:**
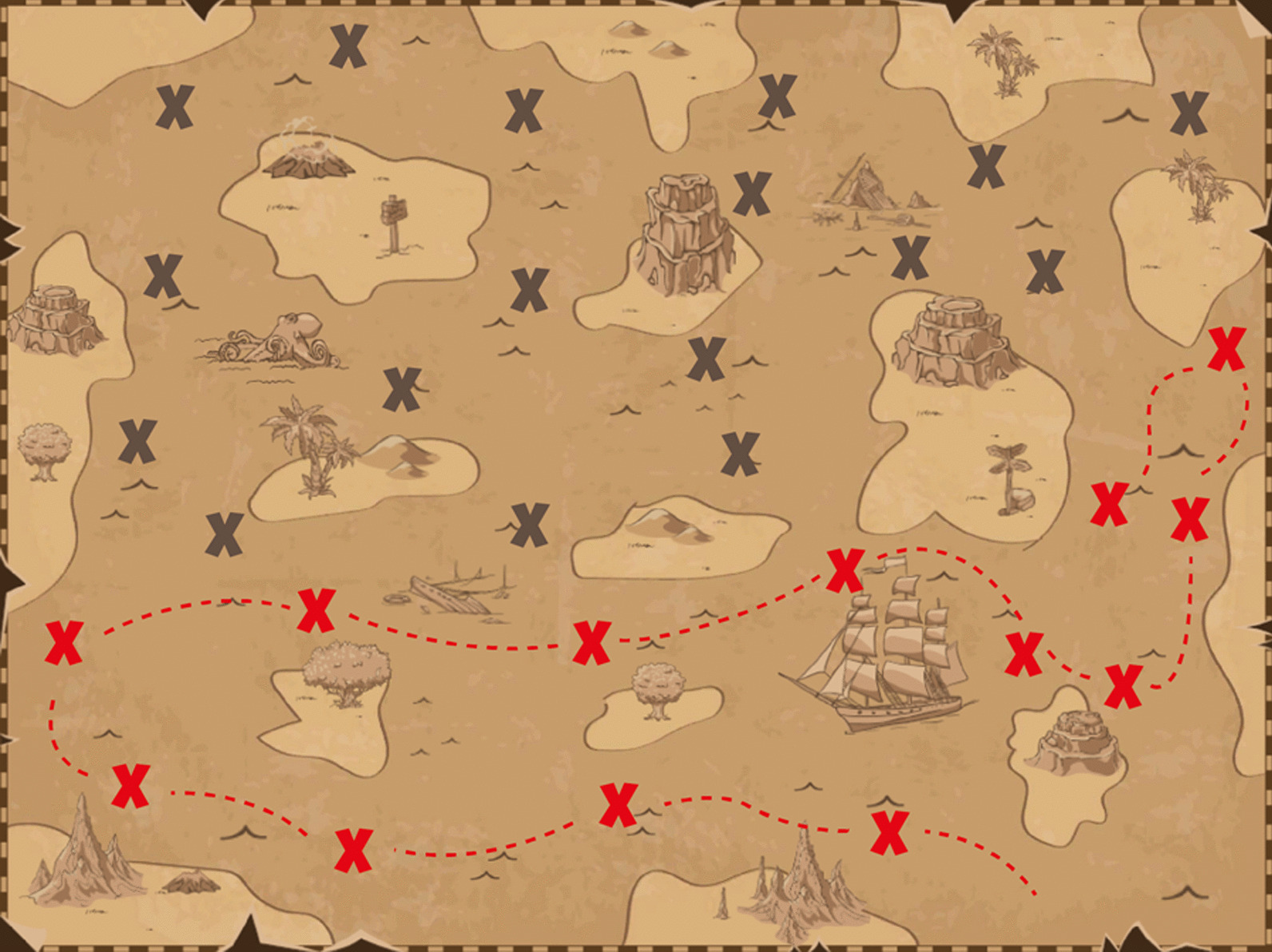
Map. The map used to illustrate the progress of the participant. Helen Galliker created images specifically for this study as part of her employment at the Center for Digital Health Interventions. The Center for Digital Health Interventions holds the copywriter to all images

As explained by BEDDA, 30 keys need to be found along the way (Fig. 4) that unlock a chest at the journey’s end (Fig. 5). These keys are hidden in bottles (Fig. 6) floating on the river on the daily trip. Besides the key, the bottle also contains the daily wisdom of the day. Each day, the participants can only find one key. Before and after each trip, BEDDA presents a map (Fig. 7) to the participants illustrating their progress (goal setting—complete a daily task [70]). The progress made is also indicated by an illustration of the number of keys collected and the days past since the journey started (gamification—progress indication or badges [70]). The participants can additionally interact with Breeze as often as they want on any given day, given that they have already completed the daily trip.

### Study design

**Fig. 8:**
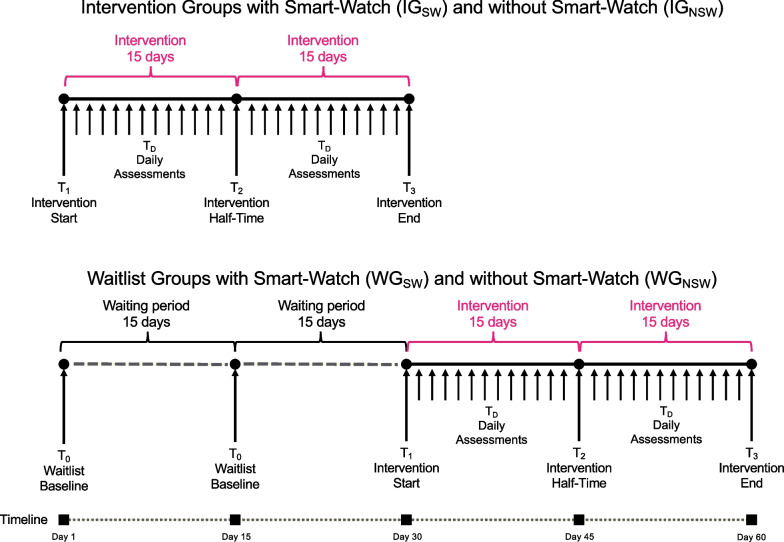
Experimental Design. Experimental design of the study. *Note*: T_0_: Assessment at baseline for the control group only. T_1_: Start intervention with start interaction and assessment. T_2_: Half-time intervention with half-time interaction and assessment. T_3_: End of intervention with final interaction and assessment. Intervention: Daily engagement in the intervention consists of interaction with the conversational agent, Breeze, providing assessments, and receiving daily wisdom. Figure created by Gisbert W. Teepe as part of his employment at the Center for Digital Health Interventions

Figure 8 shows the experimental design of the study. The study will run for 60 days, and participants will be randomly (using an algorithm on the recruitment website) allocated to one of four groups we listed in Table 1: intervention with smartwatch (IG_sw_), intervention without smartwatch (IG_nsw_), waitlist with smartwatch (WG_sw_), waitlist without smartwatch (WG_nsw_). The intervention part of the study will run for 30–45 days, depending on how many days the participant needs to finish the required 30 once-per-day guided interactions (described below). Participants allocated to the intervention group (IG_sw_ and IG_nsw_) will start the experiment first. In their first interaction (T_1_), they will receive the study information, give written informed consent via the app (type of content approved by the ethics committee), complete the initial assessment, receive a tutorial on how to use the app, and complete a training with Breeze for the first time. Participants allocated to the waitlist group (WG_sw_ and WG_nsw_) will complete the baseline assessment (T_0_) but will not yet start using the app for another 30 days. In these first 30 days, participants in the intervention group will interact daily with BEDDA. This interaction includes dialogues with the conversational agent, answering questions before and after Breeze, and conducting breathing training with Breeze. Each day, they can also choose whether they want to receive daily wisdom matching the symptom of the day. Additionally, the conversational agent moves the story further each day, and the app uses gamification elements to illustrate progress. On day 15 of the intervention, participants allocated to the intervention group will respond to the half-time assessment questions and on day 30, participants in the intervention group will respond to the final assessment.

**Table 1:** Groups included in the study

Group	Treatment	Smartwatch	Planned N
IG_sw_	Intervention	Yes	25
IG_nsw_	Intervention	No	75
WG_sw_	Waitlist	Yes	25
WG_nsw_	Waitlist	No	75

Participants assigned to the waitlist control group (WG_sw_ and WG_nsw_) will respond to baseline assessments (T_0_) on day 1 and 15 (corresponding to T_1_ and T_2_ in the intervention groups). On day 30 (T_3_ for the intervention group) the control group will start the intervention part of the study. The control group participants will be informed about the assessments, and the study starts through a notification on their smartphones. Participants in the control group will then complete the same intervention as the intervention group including the initial, half-time, and final assessment.

#### Recruitment, inclusion criteria, exclusion criteria

A highly relevant group that shows alarming rates of symptoms of subclinical [80–83] and clinical depression are undergraduate [84], graduate [85], and Ph.D. students [86]. Our study aims at this population by recruiting participants from Swiss universities with no or subclinical symptoms of depression and anxiety. To increase the diversity of our sample, we plan to recruit participants from the general population as well. We will recruit participants via mailing lists of universities, social media, and other communication channels such as flyers and advertisements. Since participants may know each other spill-over effects could occur.

Participants must be at least 18 years old, not pregnant, not diagnosed with Asthma, COPD, or other respiratory conditions, and should be willing to invest approx. five minutes of their time per day for 30 days. We will use the 9-item version of the Patient Health Questionnaire Short Version (PHQ-9, [44]) for screening purposes to determine the severity of depression. We will also use the 7-item version of the General Anxiety Disorder Questionnaire (GAD-7, [87]) to assess the severity of anxiety. Participants with scores greater than 15 (more than mild symptoms) in either screening instrument will be excluded and directly referred to a mental health hotline for the general public and mental health services of universities. We will also exclude participants responding with “several days (+1)”, “more than half the days (+2)”, or “nearly every day (+3)” to the question of the PHQ-9 assessing suicidal or self-harm ideation, and we advise the participant to seek professional help immediately. We will not use minimal cut-off scores to exclude participants because it is difficult to determine at which point a participant shows sufficient symptoms to benefit from the intervention. Additionally, we expect a selection effect due to the advertisement content focusing on improving symptoms and severity of depression (e.g., one of the outlined potential benefits is “reduce stress”).

Furthermore, we will exclude participants with a current episode of a diagnosed mood disorder (Major Depressive Disorder, Bipolar Disorder, Persistent Depressive Disorder, or a Disruptive Mood Dysregulation Disorder). The same applies to participants with other diagnosed psychiatric disorders (e.g., Generalized Anxiety Disorder, Schizophrenia, Borderline Personality Disorder). Participants currently receiving psychotherapeutic or psychopharmacological treatment can not participate in the study.

#### Enrollment and allocation

Interested respondents will complete the initial assessment using an online survey to determine whether they can participate in the study. In this survey, we will also ask eligible participants whether they would be able to pick up the smartwatch from an external institute and be willing to provide the smartwatch data for the duration of the study. Sending smartwatches to participants is not feasible due to requirements from the Cantonal Ethics Commission (Ethics Commission of the Federal State of Zurich, Switzerland) regarding anonymous data collection. Eligible respondents will be enrolled in the study and randomly allocated to either the intervention or waitlist control group using an algorithm on the website (www.bedda.me). If participants are interested in using a smartwatch, it is randomly decided whether they receive a smartwatch or not. Participants not allocated to the smartwatch group will receive instructions on downloading and installing the BEDDA app on their own. Participants assigned to the smartwatch group will receive instructions on how to book an appointment at an external organization to pick up their smartwatches. An individual from the external organization will help participants to install the app on the participantsâ€™ smartphones and enter the key of the smartwatch in the study app. We will collect no personal data during this process and cannot associate any personal data with the collected data.

Usually, blinding participants and staff is complex in most digital health intervention studies. Participants can easily realize if they are in the intervention group that uses a form of digital therapeutic or if they are in a control group (e.g., receiving standard of care or a sham intervention such as printed health information). However, we aim to address this problem by providing installation instructions for all groups after recruitment and only disclosing the intervention’s start time via a notification of BEDDA. However, the smartwatch groups will have a clear indication that they are in one of the smartwatch groups (intervention or waitlist). Still, we will neither disclose the allocation to the intervention or waitlist group to staff or participants in the smartwatch groups.

#### Daily interaction

**Fig. 9:**
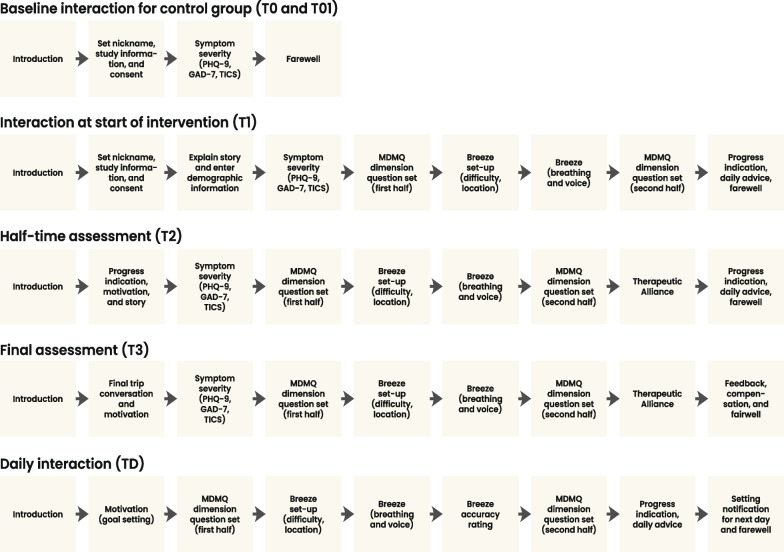
Interactions overview. Initial, daily, half-time, and final interaction within the intervention. After T_0_, the waitlist group will receive no further interaction until the intervention phase starts for this group, two weeks later with the interaction at the start of the intervention (T_1_). Note: T_0_: Assessment at baseline for control group only. T_1_: Start of intervention with start interaction and assessment. T_2_: Half-time of intervention with half-time interaction and assessment. T_3_: End of intervention with final interaction and assessment. Figure created by Gisbert W. Teepe as part of his employment at the Center for Digital Health Interventions

**Fig. 10:**
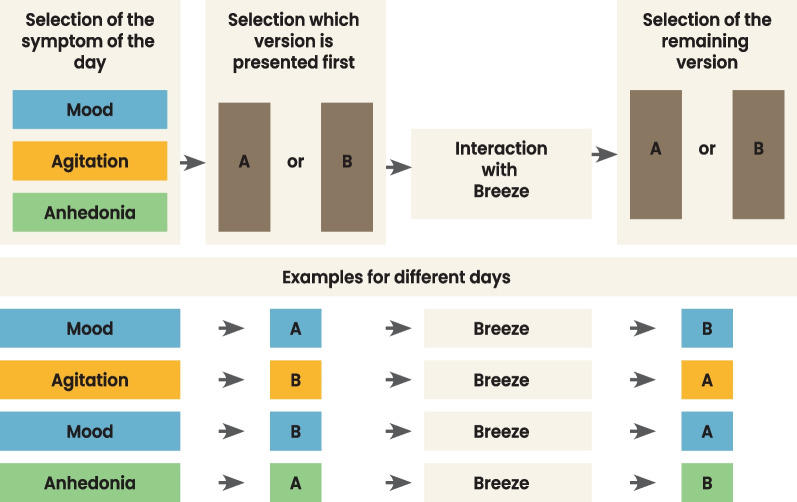
MDMQ symptom and version of the day. Selection process of which symptom is chosen for the day. First (far left, top), the app draws randomly whether Mood, Agitation, or Anhedonia is measured on a given day. Second (second box, top), the app draws randomly whether version A or version B of the symptom drawn in step one is presented first. Third (third box, top), the participant interacts with Breeze. Fourth, the app presents the remaining version of the symptom drawn of the day. In the bottom part of the figure, different draws are illustrated. The first one illustrates that on a given day Mood was randomly chosen as a symptom. The random draw determines that version A is presented first, followed by version B after interacting with Breeze. Below this, further examples are illustrated

Participants will be asked to interact with BEDDA by completing the once-per-day guided interaction (i.e., daily trip). Each daily interaction consists of different parts using the different elements of the intervention. Figure 9 illustrates such a daily interaction. First, the participants will interact with the chatbot and a treasure map showing the day's journey. Second, the participants will assess a subset of the Multidimensional Mood State Questionnaire (MDMQ) about their mood, agitation, and anhedonia [88, 89]. To reduce the burden on the participants, only one dimension of the MDMQ will be randomly selected each day. Figure 10 illustrates how the symptom of the day (e.g., symptom of the day is mood) and which version of the symptom of the day is presented in which order (e.g., Mood Version A is presented first, followed by Mood Version B after Breeze). One dimension consists of positive and negative items and has two versions with five items each. The participant will also indicate where they are conducting the breathing training (e.g., living room, office, etc.). Third, we will ask the participants to perform breathing training with Breeze. There, the app will instruct the participants to say three sentences to start the training: (1)“Lift the anchor.”, (2)“Set the sails.” (3)“Let's start today's journey.”. After this, participants will perform the breathing training. While sailing over the river, the participants will collect a bottle floating on the river. In this bottle, two items will be found the daily wisdom and one of the 30 keys. At the end of the exercise, participants will be instructed to read three sentences: (1) “Haul in the sails,” (2) “Set the anchor”, and (3) “I have finished today's journey.” Fourth, the participants will answer five different questions on the same MDMQ dimension as asked before the breathing exercise about either mood, agitation, or anhedonia. Fifth, the participants will provide a short indication of how accurately they perceived the detection of their breathing by responding to a question, adapted from Efendic et al. [90], with a seven-level response Likert scale ranging from “very inaccurate” to“very accurate”. Finally, they will be asked if they want to receive the wisdom collected on the way, showing the map of the completed journey and the number of keys they have accumulated in total.

#### Incentive mechanism

Participants will receive financial compensation for their participation if they meet certain criteria. First, participants need to interact with the app at least once per day on 30 days and complete the daily trip with the intervention. The participants can use Breeze more often on any given day, but 30 different days are needed to complete the intervention and be eligible for compensation. Second, participants need to complete the additional questions at baseline (T_0_, waitlist control group only), the start of the intervention (T_1_), the half-time (T_2_), and the final assessment (T_3_) within the once-per-day guided interaction. Third, participants must complete the intervention in 30–45 days to receive a compensation of 40 CHF. Participants needing more than 45 days will not receive any financial compensation. Forth, participants completing the intervention in 30–40 days have the chance to win additional monetary compensation in a raffle (ten times 100 CHF, ten times 200 CHF, five times 300 CHF). Participants in the smartwatch group are not eligible for this raffle but can keep the smartwatch if they complete the intervention in 30-40 days. Since we are not collecting any personal information about the participants enforcing participants with insufficient data to return the smartwatch is not feasible. However, we strongly urge the participant in the final interaction to return the smartwatch if they did not provide sufficient data.

### Measurements and assessment times

Table 2 provides an overview of the measurements used at baseline (T_0_), the intervention’s start (T_1_), half-time (T_2_), the end of the intervention (T_3_), and during each daily interaction (Daily). We collect demographic data (age, gender, type of student, highest education level, occupation, or field of study) through open and multiple-choice questions at the start of the study.

**Table 2:** Measurements used in the study at the different assessment times

Measurement	Screening	T0	T1	T2	T3	Daily	Objective
Pregnant, Resp., Card., mental disease	X	–	–	–	–	–	–
Demographics	–	–	X	–	–	–	–
*Distal outcomes*							
PHQ-9	X	X	X	X	X	–	2,3
GAD-7	X	X	X	X	X	–	2,3
TICS	–	X	X	X	X	–	2,3
*Proximal outcomes*							
MDMQ^1^	–	–	–	–	–	X	1,3
*Audio features*							
Voice commands	–	–	X^2^	X^2^	X^2^	X^2^	1,2
Breathing sounds	–	–	X^2^	X^2^	X^2^	X^2^	1,2
*Conversational agent*							
Reaction time to chatbot messages	–	X	X	X	X	X	1,2
Therapeutic alliance	–	–	–	–	X	–	3
*Qualitative feedback*							
Willingness continue using BEDDA	–	–	–	–	X	–	3
Willingness pay for BEDDA	–	–	–	–	X	–	3
Improvements suggestions	–	–	–	–	X	–	3
*Breathing exercise Breeze*							
Location	–	–	–	–	–	X	3
Perceived breathing detection accuracy	–	–	–	–	–	X	3
Accelerometer	–	–	–	–	–	X^2^	1,2
Gyroscope	–	–	–	–	–	X^2^	1,2
Magnetometer	–	–	–	–	–	X^2^	1,2
Light sensor	–	–	–	–	–	X^2^	1,2
Ambient temperature sensor	–	–	–	–	–	X^2^	1,2
Humidity sensor	–	–	–	–	–	X^2^	1,2
Pressure sensor	–	–	–	–	–	X^2^	1,2
Step counts since start	–	–	–	–	–	X^2^	1,2
Proximity sensor	–	–	–	–	–	X^2^	1,2
*Smartwatch groups*							
Physical activity	–	–	–	–	–	X^3^	1,2,3
Heart-rate	–	–	–	–	–	X^3^	1,2,3
Heart-rate variability	–	–	–	–	–	X^3^	1,2,3
Sleep duration, length of sleep phases	–	–	–	–	–	X^3^	1,2,3
Oxygen saturation	–	–	–	–	–	X^3^	1,2,3
Respiration rate	–	–	–	–	–	X^3^	1,2,3
Stress level	–	–	–	–	–	X^3^	1,2,3
Skin temperature	–	–	–	–	–	X^3^	1,2,3
Motion-based activity	–	–	–	–	–	X^3^	1,2,3
Steps	–	–	–	–	–	X^3^	1,2,3

Intervention days are repeated measures collected over each day when completing the daily trip. ^1^Each day one of three dimensions (mood, agitation, anhedonia) is used. X^2^ Only while Breeze is used. X^3^ Only in the smartwatch group

#### Voice and breathing sounds

We developed speech commands to capture commonly occurring and easily measured speech interactions. The commands are designed to be realistic in a setting outside of a study, meaning their length is similar to an interaction when giving commands to a voice assistant but also fit in the general story and setting of BEDDA. We implemented the voice commands in Breeze. To start and finish Breeze, the participants are visually instructed to say the three simple sentences before and after described above. Besides these voice interactions, we also derive the breathing sounds from Breeze. As the participant follows a guided breathing training each session with Breeze provides a set of inhalation, exhalation, and pause sounds.

#### Audio features

From the different voice commands, we will extract different features reported in related work [16, 22]. We will extract vocal folds features (i.e., source features, e.g., jitter [%], shimmer [%], tremor [Hz]), vocal tract filter features (e.g. F_1_ mean [Hz], F_2_ mean), and prosodic features (i.e., melodic, e.g., F_0_ mean, F_0_ variability, intensity [dB]). We will approach the extraction of acoustic breathing features in a more exploratory way since less related work exists regarding breathing-based biomarkers for depression. For the analysis of breathing sounds, we plan to look at the spectrograms, MFCCs, and GTCCs of the recorded breathing sounds used in previous work [91, 92].

#### Proximal and distal outcomes

Following our conceptual model, we measure proximal and distal outcomes. Proximal outcomes are the symptom changes and physiological data changes (e.g., HRV, physical activity, sleep) due to the daily interactions, while distal outcomes are the symptom severity changes over the course of the intervention. For the investigation of efficacy, we use both proximal outcomes (daily reported symptoms) and distal outcomes (symptom severity changes).

For the daily measurements (i.e., proximal outcomes) before and after interacting with Breeze, we use the Multidimensional Mood State Questionnaire (MDMQ) [88, 89], which has the three dimensions mood, agitation, or anhedonia, and two versions (Version A and B). We will infer one of the dimensions using the two versions (one before and one after interacting with the main component Breeze) at each daily interaction. For example, on a given day, the randomly chosen dimension is mood. Then, another draw determines that the questions from version A should be used to measure mood before Breeze. In turn, the questions from Version B are used to measure mood after Breeze. To ensure equal distribution of the daily drawn dimensions and versions, we use a block design consisting of the six question sets (Mood_A_, Mood_B_, Agitation_A_, Agitation_B_, Anhedonia_A_, Anhedonia_B_) and draw from this set until each was drawn once before starting with a new block for the following six days.

Our primary distal outcome is depression symptom severity, which we measure using the PHQ-9. The PHQ-9 is a subset of the Patient Health Questionnaire [44] and focuses on major depressive disorder. It is used to measure the severity of symptoms of depression in general medical and mental health settings [44]. Our secondary distal outcomes are anxiety (measured using the GAD-7) and stress (measured using the TICS). The General Anxiety Disorder-7 Questionnaire (GAD-7) is a clinically validated instrument to screen for symptom severity for the four most common anxiety disorders (Generalized Anxiety Disorder, Panic Disorder, Social Phobia, and Post Traumatic Stress Disorder) [87]. The Trier Inventory of Chronic Stress Short Version (TICS) measures all nine domains of the systemic-requirement-resource model of health [93] of an individual. It is the short version developed from the original long version of the Trier Inventory for Chronic Stress [94]. All distal outcomes are measured at T_0_, T_1_, T_2_, and T_3_ to determine symptom severity and to assess the efficacy BEDDA.

#### Other measurements

To measure the therapeutic alliance between BEDDA and the participants, we will use the Working-Alliance Inventory (WAI) [64]. We will use further qualitative open answer questions regarding BEDDA and Breeze at T_3_. Furthermore, we will ask where the participant is conducting the breathing training before starting Breeze and how accurately the breathing detection was on that day after Breeze (daily). During the interaction with Breeze, we also collect additional sensor data from the following sensors if they are present on the device: accelerometer, gyroscope, magnetometer, light sensor, ambient temperature sensor, humidity sensor, pressure sensor, step counter (since app start), and proximity sensor. The other sensors provide more information about the surroundings during the breathing training and help determine whether the person is doing the exercise correctly (e.g., steps taken during the training).

#### Smartwatches

Participants in the smartwatch groups (intervention and waitlist) will receive a smartwatch (Garmin Vívosmart 4 Smartwatch, Garmin International Inc., 1200 East 151st Street, Olathe, KS 66062, USA) at the start of the study. The measurements recorded by the smartwatch are heart-rate and heart-rate variability (via inter-beat-intervals), oxygen saturation, respiration rate, motion-based activity (3-axis accelerometer), stress level, sleep, skin temperature, and steps.

### Estimation of sample size

Sample size calculations for machine learning approaches, such as for objectives one and two, are less rigorously described compared to sample size estimations for investigation of efficacy. Therefore, we calculated the sample size needed for objective three and used this number of participants to estimate the expected correlation between features and outcomes for objectives one and two.

The third objective of our study is to investigate the efficacy of BEDDA. We operationalized this with three assessment times (at the start of the intervention, after 15 interactions, and after 30 interactions) in two groups (intervention and waitlist control). Assuming a small effect (Cohen’s d = 0.225), a power of 0.8, and an alpha level of.05, we calculated that we need at least 194 to detect an existing effect of BEDDA compared to control. Regarding our first and second objectives, we calculated that when 194 participants provide data, assuming a 0.05 alpha level and a power of 0.8, a Pearson moment correlation coefficient of 0.23 would be necessary for a significant result. Low et al. [22] reported a median number of participants for studies investigating voice changes in depression of 123 (with a range from 11 to 1688). With the estimated 194, we are above the mean but in a reasonable range.

Related work collecting data for the development of DBMs has reported a mean adherence rate of 86.6% [40]. Due to the extended assessment period, we assume a greater attrition rate. However, we also consider specific elements of our study (monetary incentives, storytelling, and gamification) that may lead to increased adherence. Considering these factors, we expect a dropout rate of approximately 20%, slightly greater than the dropout rate reported in related work [40]. Assuming this dropout rate, we aim to initially enroll 220 participants.

## Planned analysis

We plan different analyses for the different research objectives, which we will describe in this section. This description cannot be holistic for the machine learning analyses (first and second objective), as we plan to explore different machine learning algorithms. For an initial overview, we aim to provide a distribution of the daily measured proximal symptoms and the measured distal severity. The choice of algorithms will depend on the observed performance of the resulting models. Regardless of the analysis, we will split the data into a training and a hold-out testing set. Using the training set, we will train and evaluate machine learning algorithms using cross-validation. This will allow us to compare machine learning algorithms, including different feature extraction pipelines, and determine which performs best. In a final step, we will use the hold-out test set to verify the cross-validation results by evaluating the resulting models on completely unseen data.

For the first objective, we plan to investigate different supervised regression models to predict mood, agitation, and anhedonia. We aim to use the following data sources: self-reported mood, agitation, and anhedonia (MDMQ), voice sounds, breathing sounds, reaction time, and physiological smartwatch data. Due to the availability of both features (voice, breathing, and physiological features) and labels (self-reported mood, agitation, and anhedonia), we will focus on supervised machine learning models. We aim to try different machine learning algorithms, such as but not limited to multivariate linear regression models, random forest, and multilayer perceptron models. We will also explore explained variance by the voice and breathing features in self-reported and physiological data using a triangulation approach [95] to determine whether DBMS can be used as an alternative or complementary assessment for symptoms of depression.

For the second objective, we will proceed in a comparable matter. However, the dependent variables in these models are the symptom severity assessed by the PHQ-9 (depression), GAD-7 (anxiety), and TICS (stress). The independent variables remain the same (voice, breathing, physiological) with a potential extension of using the developed symptom markers from the second objective as additional independent variables. Due to only three assessment times (T_1_, T_2_, T_3_) resulting in three measurements for each participant, we will not be able to develop intra-subject models but will focus on generalizable models for the entire population. Similar to the second objective, we will also infer whether voice and breathing are complementary or alternative markers using a triangulation approach [95].

For the third objective, the estimation of efficacy, we differentiate between proximal and distal outcomes. We based this analysis on a the micro-randomized trial as introduced by Klasnja et al. [59]. However, we have the limitation that the outcomes measured are randomized and not the component. Therefore, we will use Generalized Estimating Equations (GEE; e.g., [96, 97]) to analyze the changes in proximal outcomes. Distal outcomes are the symptom severity changes as determined by the PHQ-9 (depression), GAD-7 (anxiety), and TICS (stress). As stated above in the section regarding sample size estimation, the primary distal outcome is symptom severity of depression. Due to this focus, our first analysis will be a two-way mixed ANOVA (2 groups $\times$ 3 measurement times). To investigate post-hoc, whether other factors such as belonging to the smartwatch group, age, and gender have an impact on a potential reduction of severity, we aim to use mixed effect linear regression (hierarchical groups such as intervention and smartwatch vs. no-smartwatch groups). In later analyses, we will investigate intercorrelations between the different dependent variables (PHQ-9, GAD-7, TICS) using a two-way mixed MANOVA (2 groups $\times$ 3 measurement times $\times$ 3 outcomes). We may compute additional MANOVAs comparing theoretically or empirically connected physiological outcomes to the three distal outcomes if physiological data allow such analyses.

## Discussion

In this longitudinal waitlist-control field study, we aim to collect data to improve digital health interventions addressing subclinical depression. We strive to do so by combining two main research streams in digital health: sensing (i.e., the development of DBMs to measure, understand, and predict changes in various diseases) and support (i.e., the development of standalone or blended digital therapeutics). While research in both domains has been conducted, for example, investigating the effect sizes of a just-in-time adaptive intervention [15], we aim in this study to extend these findings to a less controlled setting. This evaluation of efficacy is needed since previous work showed that the most popular and publicly available apps do not yet employ passive sensing to tailor their interventions and the majority were not evaluated in real-world randomized controlled trials [43].

Methodologically, our study may also offer further explanations regarding the underlying mechanisms of subclinical and clinical depression. Using our different analyses, we seek to understand whether voice- and breathing-based DBMs can either complement (i.e., providing explanations on a psychophysiological level) or substitute (i.e., replace used instruments describing mental state) existing approaches using self-reports to infer subclinical depression severity and symptoms of subclinical depression. By doing so, we aim to understand which physiological changes influencing speech and breathing may also drive changes in severity and symptoms of depression.

Finally, our study investigates to what degree an intervention consisting of different components (Breeze, daily wisdom, storytelling, gamification) encased by a conversational agent (BEDDA) improves symptoms of subclinical depression (proximal outcomes: mood, agitation, anhedonia), the severity of subclinical depression (primal distal outcome: PHQ-9), and the severity subclinical anxiety and stress (secondary distal outcome: GAD-7, TICS). With this, we aim to provide further evidence regarding the efficacy of standalone digital health interventions for the prevention of depression.

## Supplementary Information

**Additional file 1.** Daily Advice for Mood, Agitation, and Anhedonia.

## Data Availability

Graphics will be shared given a reasonable request for research purposes after the publication of the different planned articles. Questions regarding materials should be directed to the corresponding author.

## Abbreviations

MDD: Major depressive disorder
DBMs: Digital biomarkers
WEIRD: White, educated, industrialized, rich, democratic
AVEC: Audio/Visual Emotion Challenge Workshop
BDI-II: Beck depression inventory
BEDDA: Breathing Exercise for the Detection and Improvement of Depression and Anxiety
HRV: Heart-rate variably
CA: Conversational agent
IG: Intervention group
WG: Waitlist group
NSW: No smartwatch
SW: Smartwatch
MDMQ: Multidimensional Mood State Questionnaire
GAD-7: General Anxiety Disorder-7 Questionnaire
PHQ-9: Patient Health Questionnaire-9
TICS: Trier Inventory of Chronic Stress Short Version
WAI: Working-Alliance Inventory
GEE: Generalized Estimating Equations

## References

[1] Cuijpers P, Stringaris A, Wolpert M (2020). Treatment outcomes for depression: challenges and opportunities. Lancet Psychiatry.

[2] World Health Organisation. Special initiative for mental health (2019–2023); 2019.

[3] Jorm AF, Patten SB, Brugha TS, Mojtabai R (2017). Has increased provision of treatment reduced the prevalence of common mental disorders? Review of the evidence from four countries. World Psychiatry.

[4] Moreno-Peral P, Ángel Bellón J, Huibers MJH, Mestre JM, Garcí-López LJ, Taubner S, Rodríguez-Morejín A, Bolinski F, Sales CMD, Conejo-Cerón S. Mediators in psychological and psychoeducational interventions for the prevention of depression and anxiety. A systematic review. Clin Psychol Rev. 2020;76:101813. 10.1016/J.CPR.2020.101813. 10.1016/j.cpr.2020.101813 32045780

[5] Pfefferbaum B, North CS (2020). Mental health and the covid-19 pandemic. New Engl J Med.

[6] Brooks SK, Webster RK, Smith LE, Woodland L, Wessely S, Greenberg N, Rubin GJ (2020). The psychological impact of quarantine and how to reduce it: rapid review of the evidence. The Lancet.

[7] Berry HL, Waite TD, Dear KBG, Capon AG, Murray V. The case for systems thinking about climate change and mental health. Nat Clim Change. 2018;48(8):282–90. 10.1038/s41558-018-0102-4.

[8] Ormel J, Cuijpers P, Jorm AF, Schoevers R (2019). Prevention of depression will only succeed when it is structurally embedded and targets big determinants. World Psychiatry.

[9] Ebert DD, Cuijpers P (2018). It is time to invest in the prevention of depression. JAMA Netw Open.

[10] Kroenke K (2006). Minor depression: midway between major depression and euthymia. Ann Intern Med.

[11] Rodríguez MR, Nuevo R, Chatterji S, Ayuso-Mateos JL. Definitions and factors associated with subthreshold depressive conditions. A systematic review. BMC Psychiatry. 2012;12:1–7. 10.1186/1471-244X-12-181/TABLES/2. 10.1186/1471-244X-12-181 PMC3539957 23110575

[12] Lyness JM, Heo M, Datto CJ, Have TRT, Katz IR, Drayer R, Reynolds CF, Alexopoulos GS, Bruce ML (2006). Outcomes of minor and subsyndromal depression among elderly patients in primary care settings. Ann Intern Med.

[13] Nahum-Shani I, Smith SN, Spring BJ, Collins LM, Witkiewitz K, Tewari A, Murphy SA (2018). Just-in-time adaptive interventions (jitais) in mobile health: Key components and design principles for ongoing health behavior support. Ann Behav Med.

[14] Krebs P, Prochaska JO, Rossi JS (2010). A meta-analysis of computer-tailored interventions for health behavior change. Prev Med.

[15] Wang L, Miller LC (2020). Just-in-the-moment adaptive interventions (jitai): a meta-analytical review. Health Commun.

[16] Cummins N, Scherer S, Krajewski J, Schnieder S, Epps J, Quatieri TF (2015). A review of depression and suicide risk assessment using speech analysis. Speech Commun.

[17] Matteo DD, Fine A, Fotinos K, Rose J, Katzman M (2018). Patient willingness to consent to mobile phone data collection for mental health apps: structured questionnaire. JMIR Ment Health.

[18] Rogler LH, Malgady RG, Tryon WW (1992). Evaluation of mental health: issues of memory in the diagnostic interview schedule. J Nerv Ment Dis.

[19] van de Mortel T (2008). Faking it: social desirability response bias in self-report research. Aust J Adv Nurs.

[20] John OP, Robins RW (1994). Accuracy and bias in self-perception: individual differences in self-enhancement and the role of narcissism. J Pers Soc Psychol.

[21] Cornet VP, Holden RJ (2018). Systematic review of smartphone-based passive sensing for health and wellbeing. J Biomed Inform.

[22] Low DM, Bentley KH, Ghosh SS (2020). Automated assessment of psychiatric disorders using speech: a systematic review. Laryngosc Investig Otolaryngol.

[23] Henrich J, Heine SJ, Norenzayan A. Most people are not weird. Nature. 2010;7302(466):29. 10.1038/466029a. 10.1038/466029a 20595995

[24] Hruschka DJ, Medin DL, Rogoff B, Henrich J (2018). Pressing questions in the study of psychological and behavioral diversity. Proc Natl Acad Sci USA.

[25] Kraepelin E (1921). Manic depressive insanity and paranoia. J Nerv Ment Dis.

[26] Scherer KR (1986). Vocal affect expression. A review and a model for future research. Psychol Bull.

[27] Ellgring H, Scherer KR (1996). Vocal indicators of mood change in depression. J Nonverb Behav.

[28] Robin J, Harrison JE, Kaufman LD, Rudzicz F, Simpson W, Yancheva M (2020). Evaluation of speech-based digital biomarkers: review and recommendations. Digit Biomark.

[29] Goldsack JC, Coravos A, Bakker JP, Bent B, Dowling AV, Fitzer-Attas C, Godfrey A, Godino JG, Gujar N, Izmailova E, Manta C, Peterson B, Vandendriessche B, Wood WA, Wang KW, Dunn J. Verification, analytical validation, and clinical validation (v3): the foundation of determining fit-for-purpose for biometric monitoring technologies (biomets). npj Digit Med. 2020;1(3):1–15. 10.1038/s41746-020-0260-4. 10.1038/s41746-020-0260-4 PMC7156507 32337371

[30] Penninx BWJH, Milaneschi Y, Lamers F, Vogelzangs N (2013). Understanding the somatic consequences of depression: biological mechanisms and the role of depression symptom profile. BMC Med.

[31] Osimo EF, Baxter LJ, Lewis G, Jones PB, Khandaker GM (2019). Prevalence of low-grade inflammation in depression: a systematic review and meta-analysis of crp levels. Psychol Med.

[32] Zorn JV, Schür RR, Boks MP, Kahn RS, Joë«ls M, Vinkers CH. Cortisol stress reactivity across psychiatric disorders. A systematic review and meta-analysis. Psychoneuroendocrinology. 2017;77:25–36. 10.1016/J.PSYNEUEN.2016.11.036. 10.1016/j.psyneuen.2016.11.036 28012291

[33] Milligen BAL-v, Lamers F, Smit JH, Penninx BWJH. Physiological stress markers, mental health and objective physical function. J Psychosomat Res. 2020;133:109996. 10.1016/J.JPSYCHORES.2020.109996. 10.1016/j.jpsychores.2020.109996 32229341

[34] Trueba AF, Smith NB, Auchus RJ, Ritz T (2013). Academic exam stress and depressive mood are associated with reductions in exhaled nitric oxide in healthy individuals. Biol Psychol.

[35] Karavidas MK, Lehrer PM, Vaschillo E, Vaschillo B, Marin H, Buyske S, Malinovsky I, Radvanski D, Hassett A (2007). Preliminary results of an open label study of heart rate variability biofeedback for the treatment of major depression. Appl Psychophysiol Biofeedback.

[36] Steffen PR, Austin T, DeBarros A, Brown T (2017). The impact of resonance frequency breathing on measures of heart rate variability, blood pressure, and mood. Front Public Health.

[37] Zaccaro A, Piarulli A, Laurino M, Garbella E, Menicucci D, Neri B, Gemignani A (2018). How breath-control can change your life: a systematic review on psycho-physiological correlates of slow breathing. Front Hum Neurosci.

[38] Baumel A, Muench F, Edan S, Kane JM (2019). Objective user engagement with mental health apps: systematic search and panel-based usage analysis. J Med Internet Res.

[39] Lukic YX, Teepe GW, Fleisch E, Kowatsch T (2022). Breathing as input modality in a gameful breathing training app: development and evaluation of breeze 2. JMIR Preprints.

[40] Angel VD, Lewis S, White K, Oetzmann C, Leightley D, Oprea E, Lavelle G, Matcham F, Pace A, Mohr DC, Dobson R, Hotopf M (2022). Digital health tools for the passive monitoring of depression: a systematic review of methods. npj Digit Med.

[41] Gianfredi V, Blandi L, Cacitti S, Minelli M, Signorelli C, Amerio A, Odone A (2020). Depression and objectively measured physical activity: a systematic review and meta-analysis. Int J Environ Res Public Health.

[42] Soucy I, Provencher M, Fortier M, McFadden T (2017). Efficacy of guided self-help behavioural activation and physical activity for depression: a randomized controlled trial. Cogn Behav Therapy.

[43] Teepe GW, da Fonseca A, Kleim B, Jacobson NC, Sanabria AS, Car LT, Fleisch E, Kowatsch T (2021). Just-in-time adaptive mechanisms of popular mobile apps for individuals with depression: systematic app search and literature review. J Med Internet Res.

[44] Kroenke K, Spitzer RL, Williams JBW (2001). The phq-9. J Gen Intern Med.

[45] Beck AT, Steer RA, Brown G (1996). Beck depression inventory—ii. Psychol Assess.

[46] Mitchell AJ, Vaze A, Rao S (2009). Clinical diagnosis of depression in primary care: a meta-analysis. The Lancet.

[47] Schumann I, Schneider A, Kantert C, Löwe B, Linde K (2012). Physicians’ attitudes, diagnostic process and barriers regarding depression diagnosis in primary care a systematic review of qualitative studies. Fam Pract.

[48] Shih CH, Tomita N, Lukic YX, Reguera ÁH, Fleisch E, Kowatsch T. Breeze. Proc ACM Interact Mo Wear Ubiq Technol. 2019;3:152 . 10.1145/3369835.

[49] Lukic YX, Shih CH, Reguera AH, Cotti A, Fleisch E, Kowatsch T. Physiological responses and user feedback on a gameful breathing training app: within-subject experiment. JMIR Ser Games. 2021. 10.2196/22802. 10.2196/22802 PMC7899808 33555264

[50] Lukic YX, Klein SS, Brügger V, Keller OC, Fleisch E, Kowatsch T (2021). The impact of a gameful breathing training visualization on intrinsic experiential value, perceived effectiveness, and engagement intentions: between-subject online experiment. JMIR Ser Games.

[51] Bérubé C, Schachner T, Keller R, Fleisch E, Wangenheim FV, Barata F, Kowatsch T (2021). Voice-based conversational agents for the prevention and management of chronic and mental health conditions: systematic literature review. J Med Internet Res.

[52] Bickmore TW, Picard RW (2005). Establishing and maintaining long-term human–computer relationships. ACM Trans Comput–Hum Interact.

[53] Hauser-Ulrich S, Künzli H, Meier-Peterhans D, Kowatsch T. A smartphone-based health care chatbot to promote self-management of chronic pain (selma): pilot randomized controlled trial. JMIR mHealth uHealth. 2020. 10.2196/15806. 10.2196/15806 PMC7165314 32242820

[54] Kowatsch T, Schachner T, Harperink S, Barata F, Dittler U, Xiao G, Stanger C, Wangenheim FV, Fleisch E, Oswald H, Möller A (2021). Conversational agents as mediating social actors in chronic disease management involving health care professionals, patients, and family members: multisite single-arm feasibility study. J Med Internet Res.

[55] Anokye NK, Lord J, Fox-Rushby J (2014). Is brief advice in primary care a cost-effective way to promote physical activity?. Brit J Sports Med.

[56] Arlinghaus KR, Johnston CA (2019). The importance of creating habits and routine. Am J Lifestyle Med.

[57] van Agteren J, Iasiello M, Lo L, Bartholomaeus J, Kopsaftis Z, Carey M, Kyrios M (2021). A systematic review and meta-analysis of psychological interventions to improve mental wellbeing. Nat Hum Behav.

[58] Beun RJ, Fitrianie S, Griffioen-Both F, Spruit S, Horsch C, Lancee J, Brinkman WP (2017). Talk and tools: the best of both worlds in mobile user interfaces for e-coaching. Pers Ubiq Comput.

[59] Klasnja P, Hekler EB, Shiffman S, Boruvka A, Almirall D, Tewari A, Murphy SA (2015). Microrandomized trials: an experimental design for developing just-in-time adaptive interventions. Health Psychol.

[60] Venkatesh V, Thong JYL, Xu X (2012). Consumer acceptance and use of information technology: extending the unified theory of acceptance and use of technology. MIS Q Manag Inf Syst.

[61] Heijden HVD (2004). User acceptance of hedonic information systems. MIS Q Manag Inf Syst.

[62] Liu D, Santhanam R, Webster J. Toward meaningful engagement: a framework for design and research of gamified information systems; 2017.

[63] Venkatesh V, Morris MG, Davis GB, Davis FD (2003). User acceptance of information technology: toward a unified view. Quarterly.

[64] Horvath AO, Greenberg LS (1989). Development and validation of the working alliance inventory. J Couns Psychol.

[65] Flückiger C, Del AC, Wampold BE, Horvath AO (2018). The alliance in adult psychotherapy. A meta-analytic synthesis. Psychotherapy.

[66] Bickmore T, Gruber A, Picard R (2005). Establishing the computer–patient working alliance in automated health behavior change interventions. Patient Educ Couns.

[67] Provoost S, Lau HM, Ruwaard J, Riper H (2017). Embodied conversational agents in clinical psychology: a scoping review. J Med Internet Res.

[68] ter Stal S, Kramer LL, Tabak M, Opden Akker H, Hermens H (2020). Design features of embodied conversational agents in ehealth: a literature review. Int J Hum Comput Stud.

[69] Michie S, Ashford S, Sniehotta FF, Dombrowski SU, Bishop A, French DP (2011). A refined taxonomy of behaviour change techniques to help people change their physical activity and healthy eating behaviours: the calo-re taxonomy. Psychol Health.

[70] Knittle K, Heino M, Marques MM, Stenius M, Beattie M, Ehbrecht F, Hagger MS, Hardeman W, Hankonen N (2020). The compendium of self-enactable techniques to change and self-manage motivation and behaviour. Nat Hum Behav.

[71] Kowatsch T, Shih C-H, Lukic YX, Keller OC, Heldt K, Durrer D, Stasinaki A, Büchter D, Brogle B, Farpour-Lambert N. A playful smartphone-based self-regulation training for the prevention and treatment of child and adolescent obesity: technical feasibility and perceptions of young patients; 2021.

[72] Russell MEB, Scott AB, Boggero IA, Carlson CR (2017). Inclusion of a rest period in diaphragmatic breathing increases high frequency heart rate variability: implications for behavioral therapy. Psychophysiology.

[73] Kramer J-N, Künzler F, Mishra V, Smith SN, Kotz D, Scholz U, Fleisch E, Kowatsch T (2020). Which components of a smartphone walking app help users to reach personalized step goals? Results from an optimization trial. Ann Behav Med.

[74] Kowatsch T, Schachner T, Harperink S, Barata F, Dittler U, Xiao G, Stanger C, Wangenheim FV, Fleisch E, Oswald H, Möler A (2021). Conversational agents as mediating social actors in chronic disease management involving health care professionals, patients, and family members: multisite single-arm feasibility study. J Med Internet Res.

[75] Stieger M, Flückiger C, Rüegger D, Kowatsch T, Roberts BW, Allemand M, Urpp G (2021). Changing personality traits with the help of a digital personality change intervention. Natl Acad Sci.

[76] Stanger C, Kowatsch T, Xie H, Nahum-Shani I, Lim-Liberty F, Anderson M, Santhanam P, Kaden S, Rosenberg B (2021). A digital health intervention (sweetgoals) for young adults with type 1 diabetes: protocol for a factorial randomized trial. JMIR Res Protoc.

[77] Ollier J, Neff S, Dworschak C, Sejdiji A, Santhanam P, Keller R, Xiao G, Asisof A, Rüegger D, Bérubé C, Tomas LH, Neff J, Yao J, Alattas A, Varela-Mato V, Pitkethly A, Vara MD, Herrero R, Baños RM, Parada C, Agatheswaran RS, Villalobos V, Keller OC, Chan WS, Mishra V, Jacobson N, Stanger C, He X, von Wyl V, Weidt S, Haug S, Schaub M, Kleim B, Barth J, Witt C, Scholz U, Fleisch E, von Wangenheim F, Car LT, Müller-Riemenschneider F, Hauser-Ulrich S, Asomoza AN, Salamanca-Sanabria A, Mair JL, Kowatsch T (2021). Elena+ care for covid-19, a pandemic lifestyle care intervention: intervention design and study protocol. Front Public Health.

[78] Bickmore TW, Schulman D, Sidner CL (2011). A reusable framework for health counseling dialogue systems based on a behavioral medicine ontology. J Biomed Inform.

[79] McCusker J, Lambert SD, Cole MG, Ciampi A, Strumpf E, Freeman EE, Belzile E (2016). Activation and self-efficacy in a randomized trial of a depression self-care intervention. Health Educ Behav.

[80] Zhang B, Qi S, Liu S, Liu X, Wei X, Ming D (2021). Altered spontaneous neural activity in the precuneus, middle and superior frontal gyri, and hippocampus in college students with subclinical depression. BMC Psychiatry.

[81] Cukrowicz KC, Schlegel EF, Smith PN, Jacobs MP, Orden KAV, Paukert AL, Pettit JW, Joiner TE (2011). Suicide ideation among college students evidencing subclinical depression. J Am Coll Health.

[82] Melo-Carrillo A, Oudenhove LV, Lopez-Avila A (2012). Depressive symptoms among mexican medical students: high prevalence and the effect of a group psychoeducation intervention. J Aff Disord.

[83] Mikolajczyk RT, Maxwell AE, Ansari WE, Naydenova V, Stock C, Ilieva S, Dudziak U, Nagyova I (2008). Prevalence of depressive symptoms in university students from Germany, Denmark, Poland and Bulgaria. Soc Psychiatry Psychiatr Epidemiol.

[84] Giuntella O, Hyde K, Saccardo S, Sadoff S (2021). Lifestyle and mental health disruptions during covid-19. Proc Natl Acad Sci USA.

[85] Evans TM, Bira L, Gastelum JB, Weiss LT, Vanderford NL (2018). Evidence for a mental health crisis in graduate education. Nat Biotechnol.

[86] Levecque K, Anseel F, Beuckelaer AD, der Heyden JV, Gisle L (2017). Work organization and mental health problems in phd students. Res Policy.

[87] Spitzer RL, Kroenke K, Williams JBW, Löwe B (2006). A brief measure for assessing generalized anxiety disorder: the gad-7. Arch Intern Med.

[88] Steyer R, Schwenkmezger P, Notz P, Eid M (1994). Testtheoretische analysen des mehrdimensionalen befindlichkeitsfragebogen (mdbf). Diagnostica.

[89] Steyer R. Mdmq questionnaire (english version of mdbf).

[90] Efendić E, de Calseyde PPFMV, Evans AM. Slow response times undermine trust in algorithmic (but not human) predictions. Organ Behav Hum Decis Process. 2020;157:103–14. 10.1016/j.obhdp.2020.01.008.

[91] Chauhan J, Hu Y, Seneviratne S, Misra A, Seneviratne A, Lee Y. Breathprint: breathing acoustics-based user authentication. In: MobiSys 2017—Proceedings of the 15th annual international conference on mobile systems, applications, and services; 2017. p. 278–91. 10.1145/3081333.3081355.

[92] Valero X, Alias F (2012). Gammatone cepstral coefficients: biologically inspired features for non-speech audio classification. IEEE Trans Multimed.

[93] Petrowski K, Kliem S, Albani C, Hinz A, Brahler E (2019). Norm values and psychometric properties of the short version of the trier inventory for chronic stress (tics) in a representative German sample. PLoS ONE.

[94] Schulz P, Schlotz W, Becker P. Trierer inventar zum chronischen stress (tics) [trier inventory for chronic stress (tics)]; 2004.

[95] Tams S, Thatcher J, Hill K, Grover V, Guinea AOD (2014). Neurois-alternative or complement to existing methods? Illustrating the holistic effects of neuroscience and self-reported data in the context of technostress research. J Assoc Inf Syst.

[96] Bolger N, Laurenceau J-P Intensive longitudinal methods: an introduction to diary and experience sampling research; 2013.

[97] Walls TA, Schafer JL (2012). Models for intensive longitudinal data. Models Intens Longit Data.

[98] Voruganti T, Grunfeld E, Makuwaza T, Bender JL (2017). Web-based tools for text-based patient-provider communication in chronic conditions: scoping review. J Med Internet Res.

[99] Vecchi ND, Kenny A, Dickson-Swift V, Kidd S (2016). How digital storytelling is used in mental health: a scoping review. Int J Ment Health Nurs.

