# Supplementary material for: Development of a digital biomarker and intervention for subclinical depression: study protocol for a longitudinal waitlist control study
Source: BMC Psychol. 2023 Jun 22;11:186. doi: 10.1186/s40359-023-01215-1 (PMC10288725; doi:10.1186/s40359-023-01215-1)
Supplement: Supplementary file 1 — Additional file 1. Daily Advice for Mood, Agitation, and Anhedonia. [file 40359_2023_1215_MOESM1_ESM.pdf]

List of Daily Wisdoms (i.e., actionable advice)

Daily Wisdoms for Mood

Nr.	Title	Description	Source
1	Greet a stranger with a smile.	In our modern busy life, we don't often stop to consider the people around us. Give a stranger a nice surprise with a smile or a simple 'good morning!' and you may get one back in return.	[1, 2]
2	Positive Affirmations	Use positive self-talk to repeat feel-good phrases to yourself such as 'I am resilient', 'I can overcome challenges'. These can help rewire your brain and enhance feelings of self-esteem and self-worth	[3, 4]
3	Make a playlist of happy songs	Make a playlist of songs that bring back happy memories or just have a great beat to lift your spirits. Listen to it whenever you need a pick-me-up moment.	[5, 6, 7]
4	Viscerosensory Attention	Try to shift your attention towards and away from bodily sensations, such as heartbeat, breathing, or feelings in your stomach. Try to keep your attention focused on the different sensations for a while.	[8]
5	Emotional Imagery	Recall emotionally positive, negative, or neutral situations. Try to shift your attention shift your attention between these different situations. For example, think of a stressful exam or a conflict, a bus ride, or a beloved person. Try to keep your emotions focused on each situation for a while.	[8]
6	Facial expression	Try to express different emotions with your face and keep them for a while (e.g., angry face, neutral face, happy face).	[8]
7	Contemplative repetition	Slowly count from 1 to 10 a say a short very easily sentence. Repeat either the counting or sentence several times.	[8]
8	Active constructive responding	Share a pleasant and good experience or information with someone.	[9]
9	Exercise	Do some sort of physical activity such as going for a walk or run.	[10]
10	Social Contact	Give your best friend a call, chat with a college or fellow student over a cup of tea or spend some time with a friend or your partner.	[10]

Daily Wisdoms for Agitation

Nr.	Title	Description	Source
1	Sleep in absolute darkness.	Try to make your bedroom completely dark. Possible steps to achieve this is to use 100% opaque curtains, putting tape over power plugs that have a small light, and turn of all further light sources such as standby light.	[11]
2	Sleep in a relatively cold environment.	Try to find an appropriate sleep temperature by using the AC or opening windows. Bedroom temperature affected sleep quality more than external noise [12]. Other studies reveal that increased body and bedroom temperature can decrease sleep quality and increase wakefulness.	[13, 14, 15, 16, 17, 18]
3	Eliminate noise from your sleeping environment	Try to eliminate as much nighttime noise because it increases arousals.	[19]
4	Give yourself a prep as prep talk.	Give yourself a prep talk by thinking of what you want to achieve today and why you can do it.	[10]
5	Have a bright day.	Increase bright light exposure during the day.	[20]
6	No coffee after four.	Don't consume any type of caffeine late in the day. If you do crave a cup of coffee in the late afternoon or evening, stick with decaffeinated coffee.	[21, 22, 23].
7	Same day - same time to sleep.	Try to sleep and wake at consistent times.	[24, 25, 26, 27]
8	Don't drink and sleep.	Try to not drink any alcohol or try to drink less alcohol.	[28, 29, 30, 31, 32, 33, 34]
9	No noise, light, or mess.	Optimize your bedroom environment by trying to minimize noise (e.g., from traffic), turn of all sources of light, and tidy up your room.	[35, 36, 37, 38]
10	White noise	Listen to some white noise at bedtime.	[39]
11	Take some worry time.	Take some time before you go to bed to write down all your problems, worries and if possible, the actions or a plan to address them.	[40]
12	Not in this room. (Stimulus control)	Avoid being in the bedroom before going to bed and try to do any not sleep related activities in a different room.	[41]

Daily Wisdoms for Anhedonia

1	Master your notification settings	Try to limit your notifications and only use those that are very relevant to attend to in the moment. Health related or safety notifications should of course not be disabled.	[42, 43]
2	Take a reading break	Reading is a great way to step away from the stressors of everyday life while keeping your mind active. Choose your favorite book and take a couple of minutes for yourself to get immersed into another story	[44, 45]
3	Take time off from your phone	Keep your phone out of sight for at least the next hour to not distracted from notifications. You may choose to keep it in a cupboard or in another room	[46, 47]
4	Blessing exercise	Think about three things that today went well. If they involve another person, recognize, and thank them whenever possible	[48]
5	Savoring exercise	Think for 2-3 of something you enjoyed a lot and try to make the pleasure last as long as possible.	[49]
6	Stress acceptance	Write down a stressful event of the last week or a stressful memory that you sometimes think about. Imagine the note flies away and disappears. Finally, destroy the note.	[50]
7	Acupressure	Locate the pressure point between the base of you thumb and index finger. Press down on this point for 5 minutes. Move your thump in a circle while applying pressure. Be firm but don't press so hard that it hurts. Repeat this procedure on your other hand.	[51]
8	Unsubscribe from an email.	Observe your emails for one week and click on "unsubscribe" at the very bottom of emails that you think are not 100% relevant.	[42, 43]
9	Pay it forward	Do something kind for someone else rather than simply accepting or repaying the original good deed.	[52]
10	Mindful minutes	Here's a quick and simple mindfulness exercise [LINK] you can do to center yourself.	[53, 54, 55]

References

[1] Golland Y, Mevorach D, Levit-Binnun N. Affiliative zygomatic synchrony in co-present strangers. Scientific Reports. 2019 12;9.

[2] Gunaydin G, Oztekin H, Deniz, Karabulut H, Selin, Engin S. Minimal Social Interactions with Strangers Predict Greater Subjective Well-Being. Springer. 2021 4;22:1839-53.

[3] Falk EB, O'Donnell MB, Cascio CN, Tinney F, Kang Y, Lieberman MD, et al. Self-affirmation alters the brain's response to health messages and subsequent behavior change. Proceedings of the National Academy of Sciences of the United States of America. 2015 2;112:1977-82.

[4] Cascio CN, O'Donnell MB, Tinney FJ, Lieberman MD, Taylor SE, Strecher VJ, et al. Self-affirmation activates brain systems associated with self-related processing and reward and is reinforced by future orientation. Social Cognitive and Affective Neuroscience. 2016 4;11:621-9.

[5] Droit-Volet S, Ramos D, Bueno JLO, Bigand E. Music, emotion, and time perception: the influence of subjective emotional valence and arousal? Frontiers in Psychology. 2013;4:417.

[6] Jäncke L. Music, memory and emotion. Journal of biology. 2008;7(6):1-5.

[7] Koch K, Liu VTS, liush EZ, ethzch ETH Zürich Zürich, Zürich S, Berger T, et al. Taking Mental Health % Well-Being to the Streets: An Exploratory Evaluation of In-Vehicle Interventions in the Wild; Taking Mental Health % Well-Being to the Streets: An Exploratory Evaluation of In-Vehicle Interventions in the Wild. dlacmorg. 2021 5.

[8] Meinlschmidt G, Lee JH, Stalujanis E, Belardi A, Oh M, Jung EK, et al. Smartphone-based psychotherapeutic micro-interventions to improve mood in a real-world setting. Frontiers in Psychology. 2016 7;7:1112.

[9] Lambert NM, Gwinn AM, Baumeister RF, Strachman A, Washburn IJ, Gable SL, et al. A boost of positive affect. Journal of Social and Personal Relationships. 2013 2;30:24-43.

[10] Thayer RE, Newman JR, Mcclain TM. Self-Regulation of Mood: Strategies for Changing a Bad Mood, Raising Energy, and Reducing Tension. Journal of Personality and Social Psychology. 1994;67:910-25.

[11] Walker M. Why we sleep: The new science of sleep and dreams. Penguin UK; 2017.

[12] Libert JP, Bach V, tL C Johnson, Ehrhart J, Keller D. Relative and Combined Effects of Heat and Noise Exposure on Sleep in Humans. academicoupcom. 1991.

[13] Okamoto-Mizuno K, Tsuzuki K, Mizuno K. Effects of mild heat exposure on sleep stages and body temperature in older men. International Journal of Biometeorology. 2004;49:32-6.

[14] Okamoto-Mizuno K, Tsuzuki K, Mizuno K. Effects of humid heat exposure in later sleep segments on sleep stages and body temperature in humans. International Journal of Biometeorology. 2005 3;49:232-7.

[15] Libert J, Lenzi P. Functional Neurology and undefined sleep during prolonged heat exposure in man. PMC. 1987.

[16] Nisi JD, Ehrhart J, Galeou M, Libert JP. Influence of repeated passive body heating on subsequent night sleep in humans. European Journal of Applied Physiology and Occupational Physiology. 1989 9;59:138-45.

[17] Lack LC, Gradisar M, Van Someren EJ, Wright HR, Lushington K. The relationship between insomnia and body temperatures. Sleep medicine reviews. 2008;12(4):307-17.

[18] Valham F, Sahlin C, Stenlund H, Franklin KA. Ambient Temperature and Obstructive Sleep Apnea: Effects on Sleep, Sleep Apnea, and Morning Alertness. Sleep. 2012 4;35:513-7.

[19] Irish LA, Kline CE, Gunn HE, Buysse DJ, Hall MH. The role of sleep hygiene in promoting public health: A review of empirical evidence. Sleep medicine reviews. 2015;22:23-36.

[20] Fetveit A, Skjerve A, Bjorvatn B. Bright light treatment improves sleep in institutionalised elderly?an open trial. International Journal of Geriatric Psychiatry. 2003 6;18:520-6.

[21] Drake C, Roehrs T, Shambroom J, Roth T. Caffeine effects on sleep taken 0, 3, or 6 hours before going to bed. Journal of Clinical Sleep Medicine. 2013 11;9:1195-200.

[22] Fredholm BB, Bättig K, Holmén J, Nehlig A, Zvartau EE. Actions of caffeine in the brain with special reference to factors that contribute to its widespread use. Pharmacological reviews. 1999;51(1):83-133.

[23] Harland BF. Caffeine and nutrition. Nutrition. 2000 7;16:522-6.

[24] Dongen HPAV, Dinges DF. Investigating the interaction between the homeostatic and circadian processes of sleep-wake regulation for the prediction of waking neurobehavioural performance. Journal of Sleep Research. 2003 9;12:181-7.

[25] Giannotti F, Cortesi F, Sebastiani T, Ottaviano S. Circadian preference, sleep and daytime behaviour in adolescence. Journal of Sleep Research. 2002;11:191-9.

[26] Monk TH, Buysse DJ, Billy BD, Fletcher ME, Kennedy KS, Schlarb JE, et al. Circadian type and bed-timing regularity in 654 retired seniors: Correlations with subjective sleep measures. Sleep. 2011 2;34:235-9.

[27] Emens JS, Yuhas K, Rough J, Kochar N, Peters D, Lewy AJ. Phase angle of entrainment in morning- and evening-types under naturalistic conditions. Chronobiology International. 2009 4;26:474-93.

[28] Issa FG, Sullivan CE. Alcohol, snoring and sleep apnoea. Journal of Neurology Neurosurgery and Psychiatry. 1982;45:353-9.

[29] Carole W, Sherry L, Taasan VC, Block AJ, Boysen PG, Wynne JW. Alcohol increases sleep apnea and oxygen desaturation in asymptomatic men. The American Journal of Medicine. 1981;71:240-5.

[30] Ekman AC, Leppäluoto J, Huttunen P, Aranko K, Vakkuri O. Ethanol inhibits melatonin secretion in healthy volunteers in a dose-dependent randomized double blind cross-over study. Journal of Clinical Endocrinology and Metabolism. 1993;77:780-3.

[31] Röjdmark S, Wikner J, Adner N, Andersson DEH, Wetterberg L. Inhibition of melatonin secretion by ethanol in man. Metabolism. 1993;42:1047-51.

[32] Stevens RG, Davis S, Mirick DK, Kheifets L, Kaune W. Alcohol consumption and urinary concentration of 6-sulfatoxymelatonin in healthy women. Epidemiology. 2000;11:660-5.

[33] Wetterberg L, Aperia B, Gorelick DA, Gwirtzman HE, McGuire MT, Serafetinides EA, et al. Age, alcoholism and depression are associated with low levels of urinary melatonin. Journal of psychiatry % neuroscience : JPN. 1992 11;17:215-24.

[34] Ekman AC, Vakkuri O, Ekman M, Leppäluoto J, Ruokonen A, Knip M. Ethanol decreases nocturnal plasma levels of thyrotropin and growth hormone but not those of thyroid hormones or prolactin in man. The Journal of Clinical Endocrinology % Metabolism. 1996 7;81:2627-32.

[35] Waye KP, Clow A, Edwards S, Hucklebridge F, Rylander R. Effects of nighttime low frequency noise on the cortisol response to awakening and subjective sleep quality. Life Sciences. 2003 1;72:863-75.

[36] Halperin D. Environmental noise and sleep disturbances: A threat to health? Sleep Science. 2014;7:209-12.

[37] Bodin T, Björk J, Ardö J, Albin M. Annoyance, sleep and concentration problems due to combined traffic noise and the benefit of quiet Side. International Journal of Environmental Research and Public Health. 2015 1;12:1612-28.

[38] Lee KA, Gay CL. Can modifications to the bedroom environment improve the sleep of new parents? Two randomized controlled trials. Research in Nursing and Health. 2011 2;34:7-19.

[39] Messineo L, Taranto-Montemurro L, Sands SA, Marques MDO, Azabarzin A, Wellman DA. Broadband Sound Administration Improves Sleep Onset Latency in Healthy Subjects in a Model of Transient Insomnia. Frontiers in Neurology. 2017 12;8:718.

[40] Scullin MK, Krueger ML, Ballard HK, Pruett N, Bliwise DL. The effects of bedtime writing on difficulty falling asleep: A polysomnographic study comparing to-do lists and completed activity lists. Journal of Experimental Psychology: General. 2018 1;147:139-46.

[41] Bhat S, Pinto-Zipp G, Upadhyay H, Polos PG. "To sleep, perchance to tweet": in-bed electronic social media use and its associations with insomnia, daytime sleepiness, mood, and sleep duration in adults. Sleep Health. 2018 4;4:166-73.

[42] Eyal N. Indistractable: How to control your attention and choose your life. BenBella Books; 2019.

[43] Newport C. Digital minimalism: Choosing a focused life in a noisy world. Penguin; 2019.

[44] Canty N. Bibliotherapy: Its processes and benefits and application in clinical and developmental settings. Logos (Netherlands). 2017 11;28:32-40.

[45] Duncan S. Understanding reading for pleasure for emerging adult readers. National Research and Development Centre for Adult Literacy and Numeracy (NRDC). 2013.

[46] Fitz N, Kushlev K, Jagannathan R, Lewis T, Paliwal D, Ariely D. Batching smartphone notifications can improve well-being. Computers in Human Behavior. 2019 12;101:84-94.

[47] Lee YK, Chang CT, Lin Y, Cheng ZH. The dark side of smartphone usage: Psychological traits, compulsive behavior and technostress. Computers in Human Behavior. 2014 2;31:373-83.

[48] Emmons RA. Gratitude, subjective well-being, and the brain. The science of subjective well-being. 2008:469-89.

[49] Bryant FB, Veroff J. Savoring: A new model of positive experience. Taylor and Francis; 2017.

[50] Paredes P, Gilad-Bachrach R, Czerwinski M, Roseway A, Rowan K, Hernandez J. PopTherapy: Coping with stress through pop-culture. Proceedings - PERVASIVEHEALTH 2014: 8th International Conference on Pervasive Computing Technologies for Healthcare. 2014 7:109-17.

[51] Dibble SL, Chapman J, Mack KA, Shih AS. Acupressure for nausea: results of a pilot study. Oncology nursing forum. 2000 1;27:41-7.

[52] Hui BPH, Ng JCK, Berzaghi E, Cunningham-Amos LA, Kogan A. Rewards of kindness? A meta-analysis of the link between prosociality and well-being. Psychological Bulletin. 2020;146:1084-116.

[53] Schwind JK, McCay E, Beanlands H, Martin LS, Martin J, Binder M. Mindfulness practice as a teaching-learning strategy in higher education: A qualitative exploratory pilot study. Nurse Education Today. 2017 3;50:92-6.

[54] Wu R, Liu LL, Zhu H, Su WJ, Cao ZY, Zhong SY, et al. Brief Mindfulness Meditation Improves Emotion Processing. Frontiers in Neuroscience. 2019 10;13:1074.

[55] Howarth A, Smith JG, Perkins-Porras L, Ussher M. Effects of Brief Mindfulness-Based Interventions on Health-Related Outcomes: a Systematic Review. Mindfulness. 2019 10;10:1957-68.
